# 3-Chloro­phenyl benzoate

**DOI:** 10.1107/S1600536808022721

**Published:** 2008-07-26

**Authors:** B. Thimme Gowda, Sabine Foro, K. S. Babitha, Hartmut Fuess

**Affiliations:** a Department of Chemistry, Mangalore University, Mangalagangotri 574 199, Mangalore, India; b Institute of Materials Science, Darmstadt University of Technology, Petersenstrasse 23, D-64287 Darmstadt, Germany

## Abstract

The C=O group in the title compound, C_13_H_9_ClO_2_, is *syn* to the chloro group. The two aromatic rings are twisted by 56.88 (6)°. Adjacent mol­ecules are linked *via* weak C—H⋯O hydrogen bonding into a linear chain.

## Related literature

For previous studies, see: Gowda *et al.* (2007*a* ▶,*b* ▶,*c* ▶); Nayak & Gowda (2008 ▶).
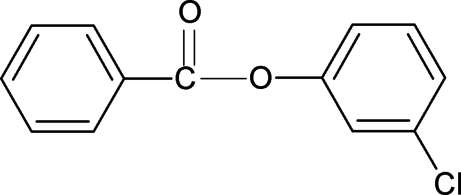


## Experimental

### 

#### Crystal data

C_13_H_9_ClO_2_
*M* *_r_* = 232.65
Triclinic, 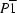

*a* = 6.0734 (6) Å
*b* = 8.389 (1) Å
*c* = 11.747 (2) Å
α = 107.89 (1)°
β = 102.98 (1)°
γ = 93.25 (1)°
*V* = 549.89 (13) Å^3^
*Z* = 2
Cu *K*α radiation
μ = 2.92 mm^−1^
*T* = 299 (2) K
0.60 × 0.55 × 0.50 mm

#### Data collection

Enraf–Nonius CAD-4 diffractometer
Absorption correction: ψ scan (North *et al.*, 1968 ▶) *T*_min_ = 0.197, *T*_max_ = 0.233
2143 measured reflections
1947 independent reflections
1872 reflections with *I* > 2σ(*I*)
*R*_int_ = 0.086
3 standard reflections frequency: 120 min intensity decay: 1.0%

#### Refinement

*R*[*F* ^2^ > 2σ(*F* ^2^)] = 0.046
*wR*(*F* ^2^) = 0.135
*S* = 1.10
1947 reflections
146 parameters
H-atom parameters constrained
Δρ_max_ = 0.30 e Å^−3^
Δρ_min_ = −0.34 e Å^−3^

### 

Data collection: *CAD-4-PC* (Enraf–Nonius, 1996 ▶); cell refinement: *CAD-4-PC*; data reduction: *REDU4* (Stoe & Cie, 1987 ▶); program(s) used to solve structure: *SHELXS97* (Sheldrick, 2008 ▶); program(s) used to refine structure: *SHELXL97* (Sheldrick, 2008 ▶); molecular graphics: *PLATON* (Spek, 2003 ▶); software used to prepare material for publication: *SHELXL97*.

## Supplementary Material

Crystal structure: contains datablocks I, global. DOI: 10.1107/S1600536808022721/ng2472sup1.cif

Structure factors: contains datablocks I. DOI: 10.1107/S1600536808022721/ng2472Isup2.hkl

Additional supplementary materials:  crystallographic information; 3D view; checkCIF report

## Figures and Tables

**Table 1:** Hydrogen-bond geometry (Å, °)

*D*—H⋯*A*	*D*—H	H⋯*A*	*D*⋯*A*	*D*—H⋯*A*
C6—H6⋯O2^i^	0.93	2.46	3.319 (3)	154

Symmetry code: (i) 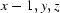.

**Fig. 1.**
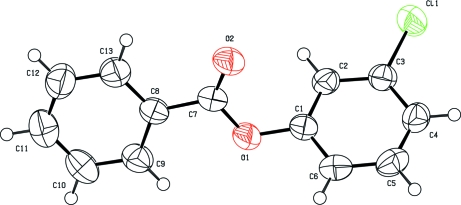
Molecular structure of the title compound, showing the atom labeling scheme. The displacement ellipsoids are drawn at the 50% probability level. The H atoms are represented as small spheres of arbitrary radii.

**Fig. 2.**
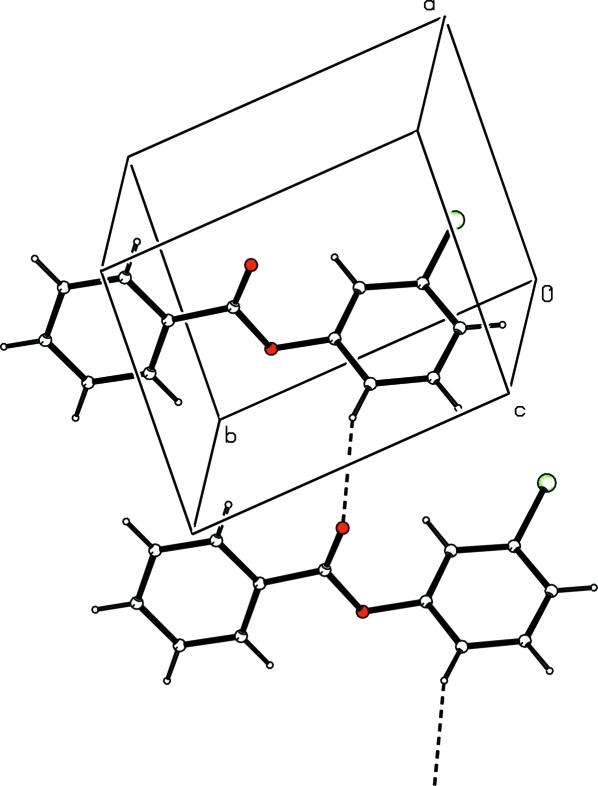
Molecular packing of the title compound.

## supplementary crystallographic information

### Comment

As part of a study of the substituent effects on the structures of aryl benzoates (Gowda *et al.*, 2007*a, b, c*), in the present work, the structure of 3-chlorophenyl benzoate (3CPBA) has been determined. The conformation of the C=O bond in 3CPBA is *syn* to the *meta*-chloro group in the phenolic benzene ring (Fig. 1), in contrast to the *anti* conformations of the C=O bond and the *meta*-methyl group in 3-methylphenyl benzoate (3MePBA) (Gowda *et al.*, 2007*a*). The bond parameters in 3CPBA are similar to those of 3MePBA (Gowda *et al.*, 2007*a*), 2,3-dichlorophenyl benzoate (23DCPBA)(Gowda *et al.*, 2007*c*), 3,4-dichlorophenyl benzoate(34DCPBA) (Gowda *et al.*, 2007*b*) and other aryl benzoates (Gowda *et al.*, 2007*a, b, c*). The dihedral angle between the benzene and benzoyl rings in 3CPBA is 56.88 (6)°, compared to the values of 79.61 (6)° in 3MePBA, 50.16 (7)° in 23DCPBA and 53.77 (5)° in 34DCPBA. The packing diagram of the crystal structure in which the molecules are connected *via* intermolecular C—H—O hydrogen bonds (Table 1) is shown in Fig. 2.

### Experimental

The title compound was prepared according to the method of Nayak & Gowda (2008). The purity of the compound was checked by determining its melting point. It was characterized by recording its infrared and NMR spectra (Nayak & Gowda, 2008). The single crystals used in X-ray diffraction studies were obtained by the slow evaporation of an ethanolic solution of the title compound at room temperature.

### Refinement

All H atoms were included in the riding-model approximation with C—H = 0.93 Å, and with *U*_iso_(H) = 1.2x*U*_eq_(C).

### Crystal data

**Table:**

C_13_H_9_ClO_2_	*Z* = 2
*M**_r_* = 232.65	*F*_000_ = 240
Triclinic, *P*1	*D*_x_ = 1.405 Mg m^−^^3^
Hall symbol: -P 1	Cu *K*α radiation λ = 1.54180 Å
*a* = 6.0734 (6) Å	Cell parameters from 25 reflections
*b* = 8.389 (1) Å	θ = 5.6–31.7º
*c* = 11.747 (2) Å	µ = 2.92 mm^−^^1^
α = 107.89 (1)º	*T* = 299 (2) K
β = 102.98 (1)º	Prism, colourless
γ = 93.25 (1)º	0.60 × 0.55 × 0.50 mm
*V* = 549.89 (13) Å^3^	

### Data collection

**Table:**

Enraf–Nonius CAD-4 diffractometer	*R*_int_ = 0.086
Radiation source: fine-focus sealed tube	θ_max_ = 66.9º
Monochromator: graphite	θ_min_ = 4.1º
*T* = 299(2) K	*h* = −7→1
ω/2θ scans	*k* = −9→9
Absorption correction: ψ scan(North *et al.*, 1968)	*l* = −13→14
*T*_min_ = 0.197, *T*_max_ = 0.233	3 standard reflections
2143 measured reflections	every 120 min
1947 independent reflections	intensity decay: 1.0%
1872 reflections with *I* > 2σ(*I*)	

### Refinement

**Table:**

Refinement on *F*^2^	Hydrogen site location: inferred from neighbouring sites
Least-squares matrix: full	H-atom parameters constrained
*R*[*F*^2^ > 2σ(*F*^2^)] = 0.046	*w* = 1/[σ^2^(*F*_o_^2^) + (0.0771*P*)^2^ + 0.1623*P*] where *P* = (*F*_o_^2^ + 2*F*_c_^2^)/3
*wR*(*F*^2^) = 0.135	(Δ/σ)_max_ = 0.002
*S* = 1.10	Δρ_max_ = 0.30 e Å^−^^3^
1947 reflections	Δρ_min_ = −0.34 e Å^−^^3^
146 parameters	Extinction correction: SHELXL97 (Sheldrick, 2008), Fc^*^=kFc[1+0.001xFc^2^λ^3^/sin(2θ)]^-1/4^
Primary atom site location: structure-invariant direct methods	Extinction coefficient: 0.149 (8)
Secondary atom site location: difference Fourier map	

### Special details

**Table:**

Geometry. All e.s.d.'s (except the e.s.d. in the dihedral angle between two l.s. planes) are estimated using the full covariance matrix. The cell e.s.d.'s are taken into account individually in the estimation of e.s.d.'s in distances, angles and torsion angles; correlations between e.s.d.'s in cell parameters are only used when they are defined by crystal symmetry. An approximate (isotropic) treatment of cell e.s.d.'s is used for estimating e.s.d.'s involving l.s. planes.
Refinement. Refinement of *F*^2^ against ALL reflections. The weighted *R*-factor *wR* and goodness of fit *S* are based on *F*^2^, conventional *R*-factors *R* are based on *F*, with *F* set to zero for negative *F*^2^. The threshold expression of *F*^2^ > σ(*F*^2^) is used only for calculating *R*-factors(gt) *etc*. and is not relevant to the choice of reflections for refinement. *R*-factors based on *F*^2^ are statistically about twice as large as those based on *F*, and *R*- factors based on ALL data will be even larger.

### Fractional atomic coordinates and isotropic or equivalent isotropic displacement parameters (Å^2^)

**Table:**

	*x*	*y*	*z*	*U*_iso_*/*U*_eq_	
Cl1	0.29134 (10)	0.17688 (7)	−0.06457 (6)	0.0678 (3)	
O1	0.2384 (2)	0.74790 (18)	0.23750 (15)	0.0565 (4)	
O2	0.5777 (2)	0.70473 (19)	0.33852 (15)	0.0587 (4)	
C1	0.1614 (3)	0.5746 (2)	0.18489 (18)	0.0474 (5)	
C2	0.2647 (3)	0.4730 (2)	0.10218 (19)	0.0477 (5)	
H2	0.3962	0.5149	0.0869	0.057*	
C3	0.1670 (3)	0.3070 (3)	0.04263 (19)	0.0482 (5)	
C4	−0.0281 (3)	0.2436 (3)	0.0636 (2)	0.0546 (5)	
H4	−0.0937	0.1321	0.0212	0.065*	
C5	−0.1247 (4)	0.3482 (3)	0.1485 (2)	0.0593 (6)	
H5	−0.2549	0.3060	0.1647	0.071*	
C6	−0.0314 (3)	0.5149 (3)	0.2099 (2)	0.0555 (5)	
H6	−0.0977	0.5853	0.2669	0.067*	
C7	0.4554 (3)	0.7994 (3)	0.30835 (17)	0.0458 (5)	
C8	0.5176 (3)	0.9840 (3)	0.34208 (17)	0.0457 (5)	
C9	0.3622 (4)	1.0902 (3)	0.3149 (2)	0.0568 (5)	
H9	0.2109	1.0464	0.2740	0.068*	
C10	0.4331 (5)	1.2610 (3)	0.3487 (2)	0.0680 (7)	
H10	0.3287	1.3323	0.3307	0.082*	
C11	0.6556 (5)	1.3268 (3)	0.4086 (2)	0.0692 (7)	
H11	0.7018	1.4423	0.4315	0.083*	
C12	0.8107 (5)	1.2214 (3)	0.4347 (2)	0.0717 (7)	
H12	0.9619	1.2658	0.4752	0.086*	
C13	0.7428 (4)	1.0512 (3)	0.4013 (2)	0.0602 (6)	
H13	0.8486	0.9804	0.4184	0.072*	

### Atomic displacement parameters (Å^2^)

**Table:**

	*U*^11^	*U*^22^	*U*^33^	*U*^12^	*U*^13^	*U*^23^
Cl1	0.0704 (4)	0.0528 (4)	0.0800 (5)	0.0084 (3)	0.0358 (3)	0.0095 (3)
O1	0.0472 (8)	0.0453 (8)	0.0692 (9)	0.0134 (6)	0.0057 (6)	0.0131 (7)
O2	0.0543 (8)	0.0553 (9)	0.0681 (10)	0.0198 (7)	0.0096 (7)	0.0247 (7)
C1	0.0436 (9)	0.0457 (11)	0.0534 (11)	0.0114 (8)	0.0092 (8)	0.0183 (9)
C2	0.0429 (10)	0.0475 (11)	0.0581 (11)	0.0066 (8)	0.0177 (8)	0.0215 (9)
C3	0.0471 (10)	0.0478 (11)	0.0560 (11)	0.0098 (8)	0.0179 (8)	0.0218 (9)
C4	0.0502 (11)	0.0492 (11)	0.0667 (13)	0.0021 (8)	0.0140 (9)	0.0241 (10)
C5	0.0447 (11)	0.0721 (15)	0.0711 (14)	0.0048 (9)	0.0210 (9)	0.0335 (12)
C6	0.0464 (10)	0.0671 (14)	0.0574 (12)	0.0162 (9)	0.0192 (9)	0.0211 (10)
C7	0.0452 (10)	0.0506 (11)	0.0456 (10)	0.0152 (8)	0.0148 (8)	0.0176 (8)
C8	0.0498 (10)	0.0493 (11)	0.0412 (9)	0.0133 (8)	0.0146 (8)	0.0161 (8)
C9	0.0574 (12)	0.0549 (13)	0.0593 (12)	0.0174 (9)	0.0124 (9)	0.0202 (10)
C10	0.0885 (17)	0.0525 (13)	0.0701 (15)	0.0255 (12)	0.0225 (12)	0.0254 (11)
C11	0.0935 (18)	0.0520 (13)	0.0634 (14)	0.0038 (12)	0.0272 (12)	0.0169 (11)
C12	0.0675 (15)	0.0653 (15)	0.0712 (15)	−0.0050 (11)	0.0129 (11)	0.0128 (12)
C13	0.0534 (12)	0.0594 (13)	0.0630 (13)	0.0109 (10)	0.0076 (9)	0.0177 (11)

### Geometric parameters (Å, °)

**Table:**

Cl1—C3	1.739 (2)	C6—H6	0.9300
O1—C7	1.356 (2)	C7—C8	1.479 (3)
O1—C1	1.399 (2)	C8—C13	1.384 (3)
O2—C7	1.195 (2)	C8—C9	1.387 (3)
C1—C2	1.374 (3)	C9—C10	1.378 (3)
C1—C6	1.374 (3)	C9—H9	0.9300
C2—C3	1.380 (3)	C10—C11	1.370 (4)
C2—H2	0.9300	C10—H10	0.9300
C3—C4	1.374 (3)	C11—C12	1.376 (4)
C4—C5	1.376 (3)	C11—H11	0.9300
C4—H4	0.9300	C12—C13	1.371 (4)
C5—C6	1.379 (3)	C12—H12	0.9300
C5—H5	0.9300	C13—H13	0.9300
			
C7—O1—C1	118.81 (14)	O2—C7—C8	125.42 (19)
C2—C1—C6	122.04 (19)	O1—C7—C8	111.50 (15)
C2—C1—O1	120.12 (18)	C13—C8—C9	119.3 (2)
C6—C1—O1	117.55 (18)	C13—C8—C7	117.74 (18)
C1—C2—C3	117.83 (17)	C9—C8—C7	122.95 (18)
C1—C2—H2	121.1	C10—C9—C8	119.6 (2)
C3—C2—H2	121.1	C10—C9—H9	120.2
C4—C3—C2	121.76 (19)	C8—C9—H9	120.2
C4—C3—Cl1	119.48 (17)	C11—C10—C9	120.7 (2)
C2—C3—Cl1	118.73 (14)	C11—C10—H10	119.7
C3—C4—C5	118.8 (2)	C9—C10—H10	119.7
C3—C4—H4	120.6	C10—C11—C12	119.8 (2)
C5—C4—H4	120.6	C10—C11—H11	120.1
C4—C5—C6	120.99 (19)	C12—C11—H11	120.1
C4—C5—H5	119.5	C13—C12—C11	120.2 (2)
C6—C5—H5	119.5	C13—C12—H12	119.9
C1—C6—C5	118.6 (2)	C11—C12—H12	119.9
C1—C6—H6	120.7	C12—C13—C8	120.4 (2)
C5—C6—H6	120.7	C12—C13—H13	119.8
O2—C7—O1	123.08 (19)	C8—C13—H13	119.8
			
C7—O1—C1—C2	−61.9 (2)	C1—O1—C7—C8	172.24 (16)
C7—O1—C1—C6	124.2 (2)	O2—C7—C8—C13	7.9 (3)
C6—C1—C2—C3	0.7 (3)	O1—C7—C8—C13	−172.14 (18)
O1—C1—C2—C3	−172.97 (16)	O2—C7—C8—C9	−173.4 (2)
C1—C2—C3—C4	0.5 (3)	O1—C7—C8—C9	6.6 (3)
C1—C2—C3—Cl1	178.84 (14)	C13—C8—C9—C10	−1.0 (3)
C2—C3—C4—C5	−1.5 (3)	C7—C8—C9—C10	−179.7 (2)
Cl1—C3—C4—C5	−179.86 (16)	C8—C9—C10—C11	0.2 (4)
C3—C4—C5—C6	1.4 (3)	C9—C10—C11—C12	0.4 (4)
C2—C1—C6—C5	−0.8 (3)	C10—C11—C12—C13	−0.2 (4)
O1—C1—C6—C5	173.01 (18)	C11—C12—C13—C8	−0.6 (4)
C4—C5—C6—C1	−0.3 (3)	C9—C8—C13—C12	1.2 (3)
C1—O1—C7—O2	−7.8 (3)	C7—C8—C13—C12	180.0 (2)

### Hydrogen-bond geometry (Å, °)

**Table:**

*D*—H···*A*	*D*—H	H···*A*	*D*···*A*	*D*—H···*A*
C6—H6···O2^i^	0.93	2.46	3.319 (3)	154

Symmetry codes: (i) *x*−1, *y*, *z*.
